# Inhibition of STAT3 signaling prevents vascular smooth muscle cell proliferation and neointima formation

**DOI:** 10.1007/s00395-012-0261-9

**Published:** 2012-03-15

**Authors:** Jan-Marcus Daniel, Jochen Dutzmann, Wiebke Bielenberg, Rebecca Widmer-Teske, Dursun Gündüz, Christian W. Hamm, Daniel G. Sedding

**Affiliations:** 1 Department of Cardiology, Justus-Liebig-University, Giessen, Germany; 2 Department of Internal Medicine I/Cardiology, Giessen University Clinic, Klinikstrasse 33, 35392 Giessen, Germany

**Keywords:** Smooth muscle cells, Neointima, Vascular remodeling, STAT3, WP1066

## Abstract

Dedifferentiation, migration, and proliferation of resident vascular smooth muscle cells (SMCs) are key components of neointima formation after vascular injury. Activation of signal transducer and activator of transcription-3 (STAT3) is suggested to be critically involved in this process, but the complex regulation of STAT3-dependent genes and the functional significance of inhibiting this pathway during the development of vascular proliferative diseases remain elusive. In this study, we demonstrate that STAT3 was activated in neointimal lesions following wire-induced injury in mice. Phosphorylation of STAT3 induced trans-activation of cyclin D1 and survivin in SMCs in vitro and in neointimal cells in vivo, thus promoting proliferation and migration of SMCs as well as reducing apoptotic cell death. WP1066, a highly potent inhibitor of STAT3 signaling, abrogated phosphorylation of STAT3 and dose-dependently inhibited the functional effects of activated STAT3 in stimulated SMCs. The local application of WP1066 via a thermosensitive pluronic F-127 gel around the dilated arteries significantly inhibited proliferation of neointimal cells and decreased the neointimal lesion size at 3 weeks after injury. Even though WP1066 application attenuated the injury-induced up-regulation of the chemokine RANTES at 6 h after injury, there was no significant effect on the accumulation of circulating cells at 1 week after injury. In conclusion, these data identify STAT3 as a key molecule for the proliferative response of SMC and neointima formation. Moreover, inhibition of STAT3 by the potent and specific compound WP1066 might represent a novel and attractive approach for the local treatment of vascular proliferative diseases.

**Electronic supplementary material:**

The online version of this article (doi:10.1007/s00395-012-0261-9) contains supplementary material, which is available to authorized users.

## Introduction

Inflammation and the subsequent activation of vascular smooth muscle cells (SMCs) are key components in the pathogenesis of vascular diseases, such as atherosclerosis, post-angioplasty restenosis, or vein bypass graft failure [12]. In response to pro-inflammatory stimuli, medial SMCs secrete various chemotactic cytokines, leading to the accumulation of leukocytes to the vessel wall [44]. During the development of vascular diseases, the chemokines RANTES (regulated upon activation, normal T-cell expressed, and secreted), stromal-cell-derived factor (SDF)-1, or monocyte chemotactic protein (MCP)-1 are considered to be key components for the recruitment of circulating cells to the vascular lesion site. The leukocytes in turn secrete various growth factors and induce the de-differentiation of quiescent and “contractile” SMCs into a “synthetic” phenotype [8]. De-differentiated SMCs are highly proliferative and migrate toward the luminal side of the vessel, thus leading to a stenotic process, which results in the development of a neointimal lesion [28, 41]. Moreover, the up-regulation of anti-apoptotic genes in neointimal SMCs leads to increased cell viability and further promotes neointima formation [29]. Therefore, a detailed understanding of the signaling mechanisms involved in the activation of SMCs is critical for the development of new and selective treatment strategies for vascular proliferative diseases [9].

The activation of the signal transducer and activator of transcription-3 (STAT3) by the Janus kinase-2 (JAK2) regulates gene expression in various biological processes, including proliferation, cell survival, and inflammation [16]. JAK2-dependent phosphorylation of STAT3 is induced in SMCs in response to various growth factors and cytokines, including platelet-derived growth factor (PDGF)-BB, angiotensin II, or interleukin (IL)-6 [27, 38]. The phosphorylation of STAT3 leads to the dimerization and the translocation of the transcription factor into the nucleus, where it trans-activates several target genes involved in proliferation and cell survival [18]. In different tumor cell lines, the up-regulation of cyclin D1 and survivin has been shown to be dependent on STAT3 activation [20, 25]. Although cyclin D1 controls the regulation of cell cycle genes and thus cell proliferation, survivin represents a member of the inhibitor of apoptosis protein (IAP) gene family that blocks activation of effector caspases in different apoptotic pathways. Even though the JAK2/STAT3 pathway has been implicated in vascular proliferative diseases, the various STAT3 target genes have barely been characterized in activated SMCs in vitro and in vivo [35, 38]. During cardiac hypertrophy and heart failure, STAT3 signaling contributes to cardioprotection by promoting cardiomyocyte survival via the interleukin (IL)-6/STAT3 axis and transcriptional regulation [3, 14]. In the process of post-conditioning, however, the cardioprotective effects of STAT3 are rather independent of gene regulation, but due to its inhibitory effect on the opening of mitochondrial permeability transition pores [4, 17].

In this study, we investigated the impact of WP1066, a highly potent inhibitor of JAK2/STAT3 signaling, on vascular SMCs and aimed to further understand the mechanisms and downstream targets of STAT3 activation. Although the parent compound AG490 only blocks phosphorylation of JAK2, WP1066 has also been described to disrupt the JAK2 protein and to block STAT3 signaling with an increase greater than ten-fold in potency compared to AG490 [13, 19]. Despite the promising in vitro results of STAT3-inhibition with AG490 or related molecules, these compounds exerted only limited efficacy in tumor models in vivo [19, 35]. In contrast, the novel and highly potent agent WP1066 is the only STAT3-targeting compound that showed potent in vivo efficacy in these models. Immunohistochemical staining of excised tumors revealed that phosphorylated (p)-STAT3 levels remained inhibited in the WP1066 treatment group up to 3 weeks after systemic WP1066 injection [19]. Thus, WP1066 represents an attractive compound that allows specific and highly potent targeting of STAT3-activation in vivo and therefore might represent an exciting novel tool for therapeutic strategies to prevent vascular proliferative diseases.

## Materials and methods

### Cell culture, detection of proliferation, migration, apoptosis, cell necrosis, and STAT3 promoter-binding

Human coronary artery SMCs were purchased from Lonza (Cologne, Germany) and cultured in optimized growth medium supplemented with 5 % FCS, 0.5 ng/ml epidermal growth factor, 2 ng/ml basic fibroblast growth factor, 5 μg/ml insulin (PromoCell, Heidelberg, Germany), according to the supplier’s protocol. Cells between passages 3 and 6 were used for all experiments.

Quantification of SMC proliferation was assessed by using a BrdU assay as previously described (Cell proliferation ELISA, Roche, Mannheim, Germany) [33]. Migration of SMC was determined in a modified Boyden chamber assay (Corning Costa Corp., Koolhovenlaan, Netherlands), apoptosis was assessed by TUNEL-based ELISA assay (cell death detection assay, Roche), and necrosis by trypan blue exclusion as previously described [33]. (*E*)-3(6-bromopyridin-2-yl)-2-cyano-*N*-((S0-1-phenylethyl)acryl-amide), or WP1066, was purchased from Sigma-Aldrich (Munich, Germany) and dissolved in dimethyl sulfoxide (DMSO, Sigma-Aldrich). STAT3 promoter-binding was assessed by the use of a STAT3-specific DNA-binding ELISA (TransAM^TM^, Active Motiv, via THP Medical Products, Wien, Austria) according to the manufacturer’s instructions.

### Preparation of cellular lysates and immunoblot analysis

Protein extraction and semi-quantitative analysis of proteins by western blotting was performed as previously described [5]. In brief, the cleared supernatant from lysates was run on polyacrylamide gel and blotted onto nitrocellulose by a commercial dry blotting system (Invitrogen, Frankfurt, Germany). After blocking, the blots were incubated with the primary antibody for 24 h at 4 °C. Primary antibodies were used for STAT3 (Cell Signaling Technology, Danvers, MA, USA), phosphorylated-(p)-STAT3 (Cell Signaling Technology), RANTES (Epitomics, Burlingame, CA, USA), and β-tubulin (Epitomics). The proteins were then detected by enhanced chemiluminescence (ECL+, Amersham) after labeling with a horseradish peroxidase-labeled secondary antibody according to the manufacturer’s instructions (GE Healthcare, Chalfont St. Giles, Great Britain).

### Real-time quantitative RT-PCR

Isolation of total RNA and synthesis of cDNA were performed using commercial kits (RNeasy and QuantiTect Rev. Transcription Kit, Qiagen, Hilden, Germany). Real-time PCR was performed on a Stratagene MX300 quantitative PCR System (Stratagene MxPro, La Jolla, CA) using the SYBR Green PCR mix (PeqLab, Erlangen, Germany). Primers were as follows: human STAT3 forward 5′-TTT GTC AGC GAT GGA GTA CG-3′, reverse 5′-GCT GCA ACT CCT CCA GTT TC-3′; mouse STAT3 forward 5′-CAA TAC CAT TGA CCT GCC GAT-3′, reverse 5′-GAG CGA CTC AAA CTG CCC T-3′; human cyclin D1 forward 5′-CCG TCC ATG CGG AAG ATC-3′, reverse 5′-ATG GCC AGC GGG AAG AC-3′; mouse cyclin D1 forward 5′-TGT GCG CCC TCC GTA TCT TAC-3′, reverse 5′-TTC TGC TCC TCA CAG ACC TCC A-3′; human survivin forward 5′-TGC TGT GGA CCC TAC TGG GTT-3′, reverse 5′-TGT CTG GGC AGA TGG CTG TTG-3′; mouse survivin forward 5′-ACC GAG AAC GAG CCT GAT TTG G-3′, reverse: 5′-GCT TTC TAT GCT CCT CTA TCG GGT T-3′; human RANTES forward 5′-ATC AAG ACA GCA CGT GGA CCT C-3′, reverse 5′-TGT GGT GTC CGA GGA ATA TGG G-3′; mouse RANTES forward 5′-TGC CGC GGG TAC CAT GAA GAT-3′, reverse 5′-TCC GAG CCA TAT GGT GAG GCG-3′; human and mouse glyceraldehyde-3-phosphate dehydrogenases (GAPDH) forward 5′-TGC ACC ACC AAC TGC TTA GC-3′, reverse 5′-GGC ATG GAC TGT GGT CAT GAG-3′. For quantification of gene expression changes, the ∆∆Ct method was used to calculate relative fold changes normalized to GAPDH. All analyses were performed in triplicate, and either the DNA template or the reverse transcriptase was omitted for control reactions.

### Mouse femoral artery injury model

All in vivo experiments were performed on adult male C57/BL6 mice purchased from Charles River (Sulzfeld, Germany). The mice were anesthetized with 150 mg/kg body weight ketaminehydrochloride (Ketanest, Pharmacia/Pfizer, Mannheim, Germany) and 0.1 mg/kg body weight xylazinehydrochloride (Rompun 2 %, Bayer, Leverkusen, Germany). The dilation of the femoral artery was performed by inserting a straight spring wire (0.38 mm in diameter; Cook, Bloomington, IN, USA) for ~10 mm towards the iliac artery, as previously described [32]. Immediately after dilation, the artery was covered with 50 μl of a 25 % thermosensitive pluronic F-127 gel containing WP1066 (200 μg/ml) or vehicle (DMSO) only. The arteries were excised at the time-points indicated in the results section. The arteries dedicated to morphometric analyses and immunohistochemistry were fixed in 4 % paraformaldehyde (PFA) and embedded in Tissue Tek OCT embedding medium (Sakura Finetek Europe B. V., Zoeterwoude, The Netherlands). The arteries were snap-frozen and stored at −80 °C until sectioning. The vessels dedicated to mRNA and protein analysis were perfused with phosphate buffered saline (PBS), microscopically rasped from adventitial as well as endothelial tissue and chopped using tissue grinder (Kimble-Chase, Vineland, NJ, USA). The mice intended for analysis of the integrity of the endothelial layer were injected with 0.5 ml of 0.5 % Evans blue dye (Sigma-Aldrich) in PBS into the tail vein. At 10 min after injection, mice were perfused with 4 % PFA and the femoral artery was excised and opened longitudinally to expose the luminal surface. All procedures involving experimental animals were approved by the Institutional Committee for Animal Research of the Giessen University and complied with the Guide for the Care and Use of Laboratory Animals (NIH publication No. 86-23, revised 1985).

### Morphometric analysis

The whole artery was cut in 6-μm serial sections, and six cross-sections from regular intervals throughout the artery were stained with hematoxylin–eosin. For morphometric analyses, Metamorph Imaging 7.0 software (Molecular Devices, Downingtown, PA, USA) was used to measure external elastic lamina, internal elastic lamina, and lumen circumference as well as to calculate medial and neointimal area.

### Immunofluorescence and immunohistochemistry

After fixation and rehydration, the slides were pre-incubated with 10 % normal goat serum (Zymed^®^ Laboratories Inc., San Francisco, CA, USA) and then incubated with antibodies against α-SMA (Sigma-Aldrich, Munich, Germany), CD31 (BD Pharmingen, Franklin Lakes, NJ, USA), CD45 (BD Pharmingen), CD68 (Serotec, Oxford, UK), STAT3 (Cell Signaling Technology, Danvers, MA, USA), von-Willebrand-factor (Dako, Glostrup, Denmark), p-STAT3 (Cell Signaling Technology), or RANTES (R&D Systems, Wiesbaden, Germany). Ensuing incubations were carried out with Alexa Fluor^®^-488 or Alexa Fluor^®^-546-coupled secondary antibodies (Molecular Probes, Eugene, Oregon, USA) and counterstained with nuclear 4.6-diamidino-2-phenylindole (DAPI) (Linaris, Wertheim, Germany). Monoclonal antibodies to α-SMA were labeled directly with Cy3. The detection of STAT3 and p-STAT3 required membrane permeabilization using Triton X-100 (0.2 %, Bio-Rad, Munich, Germany) or heat-mediated antigen retrieval by incubating the slides in sodium citrate buffer for 10 min at 90 °C. Staining for proliferating cell nuclear antigen (PCNA) was performed with a PCNA staining kit (Invitrogen, Frankfurt, Germany). The number of apoptotic SMCs was quantified by terminal deoxynucleotidyl transferase-mediated dUTP nick end-labeling (TUNEL) according to the supplier’s instructions (in situ cell death detection kit, Roche). Re-endothelialization was quantified by estimating the lumen coverage on a scale from 0 to 6 (0, no coverage; 6 complete coverage). Negative controls were conducted by substituting the primary antibody with an appropriate species- and isotype-matched control antibody (Santa Cruz Biotechnology, Santa Cruz, CA, USA).

### Statistical analysis

Data between the study groups were analyzed with 1-way ANOVA (Systat, Erkrath, Germany) followed by pair-wise multi-comparison using the Holm-Sidak method. Statistically significant differences in normally distributed variables between two groups were determined by using the Student’s *t* test. A probability value <0.05 was considered statistically significant for all comparisons.

## Results

### STAT3 is phosphorylated and up-regulated in the developing neointimal lesion

**Fig. 1:**
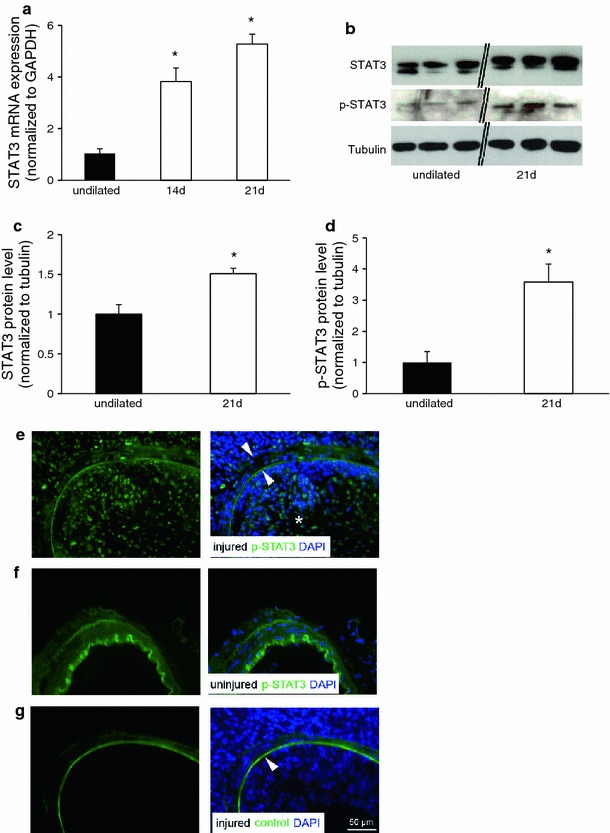
Phosphorylation and up-regulation of STAT3 during neointima formation. **a** Real-time PCR revealed a significant up-regulation of STAT3 mRNA levels in the dilated artery at 2 and 3 weeks after dilation (*P < 0.05, *n* = 4). **b**–**d** Western blotting experiments showed a significant up-regulation of total STAT3 (1.5 fold) and phosphorylated (p)-STAT3 (3.6 fold) at 3 weeks after dilation. **e** Immunohistochemical staining for p-STAT3 at 3 weeks after dilation revealed that the transcription factor is activated and located within the nucleus during the development of a neointimal lesion. The neointimal lesion (*asterisk*) and the medial layer are indicated (*arrowheads* indicate the auto-fluorescent internal and external elastic laminae defining the medial layer). **f** In contrast, there was virtually no p-STAT3 detectable in uninjured arteries. **g** A species- and isotype-matched control antibody was used as a negative control. The internal elastic lamina shows strong auto-fluorescence (*arrowhead*)

In a mouse model of wire-induced injury of the femoral artery, a neointimal lesion usually develops within 14–21 days after dilation [10]. Real-time PCR of the neointimal tissue revealed significantly higher levels of STAT3 mRNA at both 14 days and 21 days after dilation compared to non-dilated control arteries (4.28 ± 0.39-fold up-regulation at 21 days after injury compared to uninjured arteries, *n* = 6, *P < 0.05, Fig. 1a). The up-regulation of total STAT3 protein levels as well as an increase in p-STAT3 was confirmed by western blotting of excised neointimal lesions compared to uninjured arteries (*n* = 6, *P < 0.05, Fig. 1b). The increase in p-STAT3 (3.6 folds) was more prominent than the increase in total STAT3 protein levels (1.5 folds) in the developing neointimal lesion (Fig. 1c, d). Immunohistochemical staining for p-STAT3 confirmed the western blotting results and revealed significant amounts of the activated form of the transcription factor within the nuclei of neointimal cells (Fig. 1e). In contrast, p-STAT3 was hardly expressed in non-injured arteries (Fig. 1f). Importantly, co-staining of p-STAT3 with smooth muscle marker proteins (α-smooth muscle actin) revealed high expression levels of p-STAT3 in neointimal SMCs and also of smooth muscle-like cells in the adventitial layer (Suppl. Fig. 1a). Within the neointimal lesion, however, STAT3/p-STAT3 expression was also detectable in areas with distinctive staining for monocytes/macrophages (CD68), especially within the medial layer (Suppl. Fig. 1b). As a negative control, an appropriate species- and isotype-matched control antibody was used (Fig. 1f).

### Survivin and cyclin D1 are STAT3-dependent target genes in stimulated SMCs in vitro and in neointimal cells in vivo

**Fig. 2:**
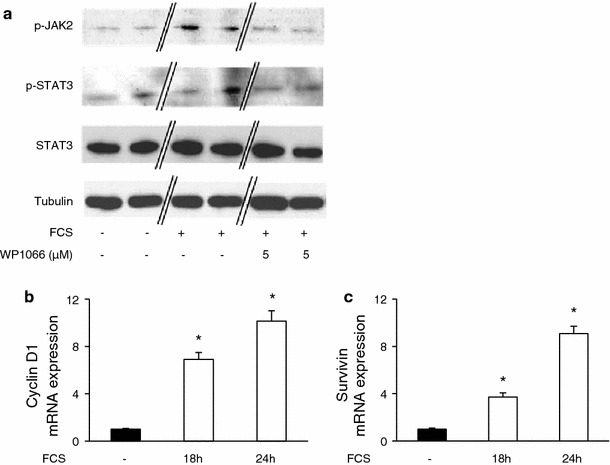
Activation and up-regulation of STAT3 and its target genes in stimulated SMCs. **a** At 10 min after stimulation of SMCs with growth medium supplemented with FCS, JAK2, and STAT3 were found to be phosphorylated using western blotting. The phosphorylation of JAK2 and STAT3 could be prevented by WP1066. **b**, **c** SMCs were incubated in growth medium for 18 or 24 h and cyclin D1 as well as survivin mRNA levels were found to be up-regulated using real-time PCR (*P < 0.05, *n* = 4)

**Fig. 3:**
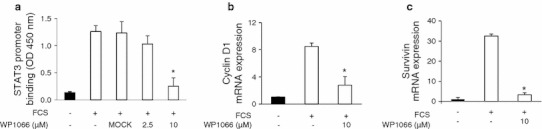
WP1066 abrogates nuclear binding of STAT3 and expression of its target genes. **a** SMCs were grown in the absence or presence of different concentrations of WP1066 for 24 h and STAT3 promoter-binding was assessed. **b**, **c** Real-time PCR revealed that mRNA levels of cyclin D1 and survivin were found to be abrogated in the presence of WP1066 (*P < 0.05, *n* = 4)

Mitogenic stimulation of SMCs resulted in phosphorylation of STAT3 in vitro, as shown by western blotting analysis at 10 min after treatment. Concomitantly, we detected phosphorylation of JAK2 after stimulation of the cells, which is a prerequisite for the subsequent phosphorylation of cytosolic STAT3 proteins. The phosphorylation of JAK2/STAT3 could be prevented following the administration of WP1066, a specific and potent STAT3 inhibitor (Fig. 2a) [14]. At 4 and 8 h after stimulation, we detected a significant up-regulation of STAT3 mRNA levels in SMCs in vitro. This up-regulation of STAT3 could be confirmed on the protein level at 8 and 12 h after stimulation (Suppl. Fig. 2). WP1066 potently prevented the phosphorylation of STAT3 but had no effect on the regulation of STAT3 mRNA or protein levels at early time-points after stimulation (data not shown). Since phosphorylation of STAT3 as the principle mechanism of STAT3 signaling is critically involved in proliferation and cell survival [16], we aimed to identify target genes of p-STAT3 that mediate the functional effects of the transcription factor in SMCs. We found the mRNA levels of both cyclin D1 and survivin to be significantly up-regulated in stimulated SMCs at 18 and 24 h and within the neointimal lesion of dilated arteries, as determined by real-time PCR (Fig. 2b, c and Suppl. Fig. 3). A DNA-binding ELISA assay revealed an enhanced binding of STAT3 to corresponding promoter-binding sites in SMCs after stimulation with growth medium (Fig. 3a). Importantly, the specific STAT3 inhibitor WP1066 dose-dependently prevented DNA-binding of STAT3 to its promoter regions. At WP1066 concentrations of 10 μM, the promoter-binding of STAT3 was almost completely prevented (Fig. 3a). Consequently, the trans-activation of the STAT3 target genes cyclin D1 and survivin was effectively blocked by WP1066 in growth medium-stimulated SMC, as determined by qPCR at 24 h after stimulation (Fig. 3b, c).

### WP1066 abrogates the functional effects of growth medium stimulation on SMC

**Fig. 4:**
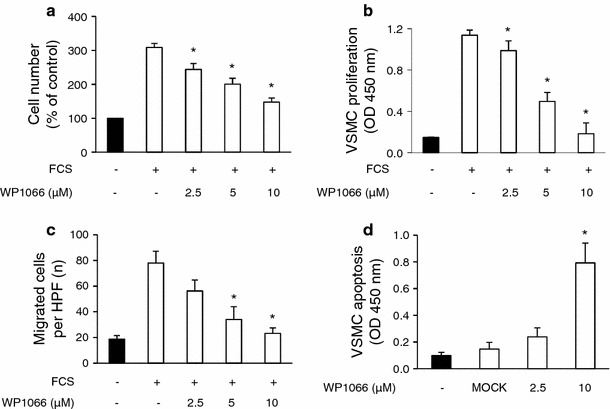
WP1066 influences proliferation, migration and apoptosis of stimulated SMCs. **a** SMCs were incubated with growth medium in the absence or presence of different concentrations of WP1066 and total cell number was evaluated after 48 h (*P < 0.05, *n* = 4). **b** SMCs were incubated with growth medium in the absence or presence of different concentrations of WP1066 for 24 h in the presence of BrdU. SMC proliferation is expressed as mean OD450 ± SEM as determined by anti-BrdU ELISA (*P < 0.05, *n* = 4). **c** SMCs were added to the upper side of gelatin-coated tissue culture inserts and allowed to migrate for 6 h in the presence or absence of PDGF (20 ng/ml) or different concentrations of WP1066. After microscopic evaluation of inserts, the number of migrated cells was expressed as cells per high power field (cells/HPF) (*P < 0.05, *n* = 4). **d** SMCs were incubated in basal medium in the absence or presence of different concentrations of WP1066 for 24 h, and apoptosis of SMCs was evaluated by a TUNEL-based cell death detection ELISA (*P < 0.05, *n* = 4)

To evaluate the functional effects of STAT3 inhibition on proliferation, migration, and apoptosis of SMCs, human coronary SMCs were incubated with growth medium in the presence or absence of different concentrations of WP1066. Application of WP1066 dose-dependently prevented the increase in SMC cell numbers after incubation with growth medium for 48 h (cell numbers increased to 308.5 ± 12 % vs. 147.5 ± 12 % at 10 μM WP1066; *n* = 4; *P < 0.05; Fig. 4a). The results from a BrdU incorporation assay showed that proliferation of SMCs was dose-dependently attenuated and almost completely prevented at concentrations of 10 μM WP1066 (Fig. 4b). Moreover, WP1066 dose-dependently inhibited the migration of SMCs as determined by a modified Boyden chamber assay (78.08 ± 9.1 vs. 23.17 ± 4.33 cells/high power field at 10 μM WP1066; *n* = 4; *P < 0.05; Fig. 4c). The reduction in cell numbers was due to both an inhibition of SMC proliferation and an increase in the percentage of apoptotic (TUNEL+) SMCs (OD 0.099 ± 0.024 vs. OD 0.793 ± 0.148 at 10 μM WP1066;* n* = 4; *P < 0.05; Fig. 4d). The increase in apoptotic cell death of SMCs was observed under basal conditions as well as under mitogenic conditions (Suppl. Fig. 4a). There was no effect of WP1066 on cell necrosis at 2.5, 5, and 10 μM, as determined by Trypan blue exclusion. However, we observed toxic effects of WP1066 at 20 μM or higher concentrations, which significantly increased necrotic cell death of SMCs in vitro (Suppl. Fig. 4b).

### WP1066 prevents neointima formation in vivo

**Fig. 5:**
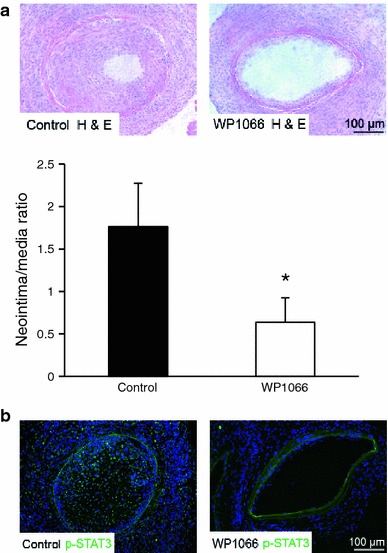
WP1066 prevents neointima formation in vivo. **a** Representative cross-sections of femoral arteries from control mice (*left*) or mice treated with WP1066 (*right*) are shown at 3 weeks after dilation. The intima/media (I/M) ratio from control mice (*left*) or mice treated with WP1066 (*right*) was quantified at 3 weeks after injury (*n* = 6, *P < 0.05). **b** Application of WP1066 (*right*) markedly reduced staining for p-STAT3 at 3 weeks after injury compared to the control group (*left*)

To determine whether our in vitro results on the effects of WP1066 were reproducible in vivo, we dilated the femoral artery and applied 50 μl of a thermosensitive pluronic F-127 gel containing WP1066 (200 μg/ml) or vehicle control around the dilated region of the artery [32]. At 21 days after wire-induced injury, a significant concentric neointimal lesion had developed, whereas the medial layer could be clearly defined by the internal and external elastic laminae (Fig. 5a). In WP1066-treated mice, the neointimal lesion size was significantly reduced at 21 days after dilation of the artery (NI/media ratio 0.64 ± 0.29 vs. 1.76 ± 0.51 in the control group, *n* = 6; *P < 0.05; Fig. 5a). In accordance with our in vitro *data*, we detected a reduced expression p-STAT3 within the vessel wall of WP1066 treated mice, whereas the neointimal lesion in the vehicle control showed a robust expression of p-STAT3 within the neointimal cellular mass (Fig 5b).

### WP1066 inhibits proliferation and increases apoptotic rates of neointimal SMC

**Fig. 6:**
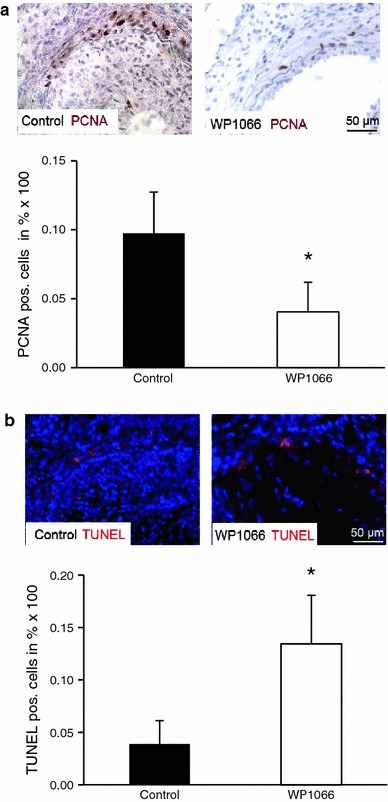
WP1066 inhibits proliferation and increases apoptosis of vascular cells in vivo. **a** Representative cross-sections of femoral arteries from control mice (*left*) or mice with WP1066 application (*right*) at 3 weeks after dilation are stained for PCNA (*brown*) or hematoxylin (*blue*). The number of proliferating (PCNA positive) cells within the neointima and media was determined by dividing the number of PCNA-positive cells per section by the total cell number of cells per section (*n* = 6, *P < 0.05). **b** Representative cross-sections of femoral arteries from control mice (*left*) or mice with WP1066 application (*right*) at 3 weeks after dilation are stained for TUNEL (*red*) or DAPI (*blue*). The number of apoptotic cells within the neointima and media was determined by dividing the number of TUNEL positive cells per section by the total cell number per section (*n* = 6, *P < 0.05)

Further immunohistochemical evaluation of the neointimal lesions revealed that WP1066 reduced neointima formation by inhibiting SMC proliferation and increasing the percentage of apoptotic cells within the vascular wall. In the WP1066-treated group, the proliferation of neointimal and medial cells was significantly reduced at 21 days after injury, as determined by the percentage of PCNA-positive cells within the vascular wall (9.7 ± 3 % vs. 4.0 ± 2.16 % PCNA-positive cells in arteries from mice treated with WP1066, *n* = 6, *P < 0.05; Fig. 6a).

In contrast, the percentage of apoptotic cells was significantly increased in the dilated arteries after treatment with WP1066, as determined by quantification of TUNEL-positive cells in situ (3.9 ± 2.22 % vs. 13.4 ± 4.64 % TUNEL-positive cells in arteries from mice treated with WP1066, *n* = 6, *P < 0.05; Fig. 6b). Re-endothelialization of the dilated arteries was mostly complete and did not show a significant difference between the vehicle-treated group and the WP1066-treated group at 21 days after injury (Suppl. Fig. 5). In addition, there were no deleterious effects of local WP1066 application on the intact endothelial layer of uninjured arteries (Suppl. Fig 6).

### Effects of WP1066 on RANTES expression and leukocyte accumulation after vascular injury

**Fig. 7:**
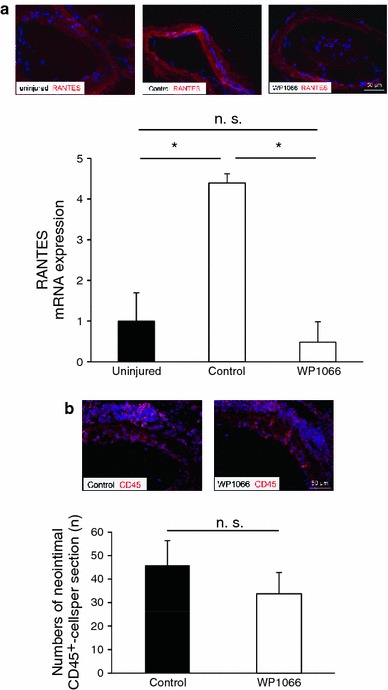
WP1066 prevents up-regulation of RANTES after vascular injury but does not significantly reduce the accumulation of circulating cells to the lesion. **a** Representative cross-sections of femoral arteries at 6 h after dilation show that the up-regulation of RANTES after injury is inhibited by WP1066. Real-time PCR of femoral artery extracts confirm the up-regulation of RANTES on the mRNA level at 6 h after injury and the prevention by WP1066. **b** In contrast, the accumulation of leukocytes at 1 week after dilation did not show a significant difference between control mice (*left*) and mice after WP1066 application (*right*) (*n* = 6, *P < 0.05)

The chemokine RANTES mediates leukocyte accumulation and its regulation after vascular injury has been described to be dependent on STAT3 signaling [22]. At 6 h after dilation, we found RANTES mRNA levels to be significantly up-regulated compared to uninjured femoral arteries, as determined by qPCR of extracted vessels. This profound up-regulation of RANTES on the mRNA level could be confirmed by immunohistochemical staining on the protein level. Importantly, application of WP1066 immediately after injury prevented transcription of RANTES (*P* < 0.05, *n* = 6) and also led to reduced immunoreactivity of RANTES within the vessel wall (Fig. 7a). At 6 h after injury, we found only low numbers of leukocytes in the peri-vascular tissue and very few adhering at the arterial lesion site (data not shown).

The effects of WP1066 on the accumulation of leukocytes were further investigated at 1 week after injury (the time-point of the peak inflammatory response in this injury model). Indeed, in WP1066-treated mice, we found a slightly reduced accumulation of CD45-positive cells at the arterial lesion site. However, this effect was not statistically significant (Fig 7b). Importantly, peripheral blood cell counts at 1 week after injury did not indicate any significant difference between the vehicle-treated group and the WP1066-treated group in regard to circulating leukocytes, erythrocytes, or platelets (Table 1, Suppl. Fig. 7).

**Table 1:** Blood tests were performed at 1 week after injury, in order to analyze the systemic effects of WP1066

	Control	WP1066	*P* value
*N*	5	6	
Leukocytes (10^9^/L)	3.72 ± 1.14	3.28 ± 0.97	0.511
Erythrocytes (10^12^/L)	8.94 ± 0.55	8.97 ± 0.69	0.537
Hemoglobin (g/L)	137.60 ± 9.40	131.67 ± 12.52	0.406
Hematokrit (L/L)	0.44 ± 0.04	0.44 ± 0.04	0.748
Mean corpuscular volume (fL)	49.60 ± 2.41	48.67 ± 1.63	0.464
Mean corpuscular hemoglobin (pg)	15.40 ± 0.85	14.68 ± 0.44	0.354
Mean corpuscular hemoglobin concentration (g/dL)	30.96 ± 1.51	30.17 ± 0.92	0.239
Platelets (10^9^/L)	272.20 ± 89.19	513.17 ± 238.72	0.330
Eosinophils (10^9^/L)	0.03 ± 0.06	0.01 ± 0.03	0.762
Segmented Neutrophils (10^9^/L)	0.74 ± 0.41	0.54 ± 0.35	0.824
Basophils (10^9^/L)	0.00 ± 0.00	0.00 ± 0.00	1.000
Lymphocytes (10^9^/L)	2.98 ± 0.77	2.56 ± 0.88	0.760
Monocytes (10^9^/L)	0.07 ± 0.14	0.06 ± 0.05	0.352
Creatinine (mg/dL)	0.10 ± 0.00	0.09 ± 0.04	0.762
Albumin (g/L)	22.75 ± 2.50	22.17 ± 3.06	0.760
Lactate dehydrogenase (U/L)	523.00 ± 97.97	458.40 ± 85.36	0.114
Aspartate transaminase (U/L)	142.00 ± 60.65	172.17 ± 61.93	0.469
Alanine transaminase (U/L)	25.50 ± 1.91	25.00 ± 4.15	0.830

There was no significant difference between vehicle (DMSO) and WP1066 treated mice

## Discussion

STAT3 is phosphorylated in response to growth factors and cytokines in a variety of proliferating cell types, including SMCs in vitro [16, 35]. We now provide the first in vivo data on elevated p-STAT3 levels in dilated mouse arteries, leading to a profound up-regulation of cyclin D1 and survivin. Furthermore, we characterize the functional effects of STAT3 phosphorylation in stimulated SMCs and show that the specific inhibitor WP1066 dose-dependently blocks STAT3 activation and the trans-activation of its target genes cyclin D1 and survivin. As a proof of concept that phosphorylation of STAT3 is crucial for the development of vascular proliferative diseases, we show that the specific STAT3 inhibitor WP1066 effectively prevents neointimal lesion formation after wire-induced injury.

Proliferation, migration, and apoptosis of SMC are all fundamental components in the development of a neointimal lesion [9, 12]. In the process of neointima formation, the STAT3 target gene cyclin D1 does not only represent a key regulator of cell cycle genes but also of SMC migration and thus cell motility [21, 38]. Moreover, it has recently been shown that anti-apoptotic genes are up-regulated in neointimal SMCs and that blockade of these anti-apoptotic genes is effective in preventing neointima formation [29]. Interestingly, survivin has been identified as a critical regulator of cell survival in SMC in vivo, and disrupting the survivin pathway effectively suppresses neointima formation after vascular injury [2]. Hence, WP1066 inhibits neointima formation by two additive mechanisms: there is a beneficial effect from reducing the proliferation and migration of SMCs via preventing the expression of cyclin D1. Furthermore, the neointimal cellular mass is reduced by increased apoptosis of SMCs due to a reduced survivin expression. Although our analysis of STAT3-dependent target genes is limited, we presume that cyclin D1 and survivin, acting via different mechanisms, represent two key components with a high functional significance on neointima formation.

In the recent reports, STAT3 activation could be linked to functional effects on neointimal cells, and inhibition of STAT3 signaling by AG490 was shown to antagonize these effects [35, 36]. In our experiments, we used WP1066, a STAT3 inhibitor that is significantly more active and potent compared to its parent compound AG490 [11, 13]. Even though AG490 routinely exerts potent in vitro activity, AG490 has not consistently been demonstrated to have a potent in vivo effect in animal models, except when applied in very high (and potentially toxic) doses [19, 35]. In contrast, WP1066 has been shown to specifically prevent STAT3 phosphorylation at low doses in tumor models up to 3 weeks after the last injection of the inhibitor [19]. In addition to its high potency, WP1066 thus exerts its specific effects on vascular SMCs over a long time-period after arterial injury. Both of these characteristics are absolutely mandatory for the successful use of a drug to inhibit neointima formation in the clinical setting.

Inflammation is another key component of neointimal lesion formation [15, 34]. The denudation of the endothelium and the cell death of medial SMCs after vascular injury are followed by the secretion of cytokines and chemokines, leading to the recruitment of leukocytes to the vascular lesion [40]. Importantly, the secretion of the chemokine RANTES from SMCs at several hours after vascular injury is dependent on STAT3 expression [22]. Even though we could show that inhibiting STAT3 signaling prevents the up-regulation of RANTES at 6 h after injury, the effect on the accumulation of leukocytes at 1 week after dilation was only moderate. A complex network of cytokines and chemokines is involved in the recruitment of circulating cells, including RANTES, MCP-1, and SDF-1α (CXCL12) [24, 43]. Although SDF-1α and RANTES are highly expressed within the medial layer of the dilated arteries at 6 h after injury, the expression of SDF-1α and RANTES within the medial layer rapidly decreases at later time-points after injury [22, 42]. Instead, platelets adhering to the disrupted endothelial layer represent a major source of chemokines and have been shown to carry high amounts of RANTES mRNA [6, 30]. The inhibition of platelet-derived RANTES by systemic injection of specific RANTES receptor antagonists has been shown to reduce macrophage infiltration and neointimal lesion formation [31]. However, in our model, WP1066 was applied around the adventitial layer and thus did not directly target platelet-derived RANTES. Therefore, the effects of WP1066 on the initial secretion of RANTES by SMCs do not significantly reduce the accumulation of leukocytes in our model. Instead, we provide evidence that WP1066 impedes the proliferative response of vascular SMCs by inhibiting phosphorylation of STAT3 and thus trans-activation of cyclin D1 and survivin. This is further supported by the detection of WP1066 specific effects for up to 3 weeks after application of the inhibitor in a tumor model [19]. Our immunohistochemical studies confirm that WP1066 was very effective in reducing phosphorylation of STAT3 and the proliferative response of SMCs within the vessel wall for at least 3 weeks after injury. Interestingly, we also detected adventitial cells expressing p-STAT3 during the process of neointima formation (Suppl. Fig 1). Since clonal expansion of (myo-)fibroblasts and also peri-vascular progenitor cells have been described to contribute to neointimal lesion formation, it is very likely that WP1066 does not only inhibit proliferation/migration of genuine SMCs but also of adventitial (progenitor-)cells [23].

Even though the process of re-endothelialization was not affected at 21 days after injury, there is accumulating evidence that STAT3 also influences the proliferation of endothelial cells [1, 39]. Therefore, application of WP1066 could possibly delay re-endothelialization of the dilated vessel, so that a prolonged anti-thrombotic therapy might be necessary to abrogate this potential side effect of WP1066 [9]. However, our data indicate that application of WP1066 does not impair endothelial integrity in neighboring or uninjured vessel areas. Thus, WP1066 holds promise to emerge as a promising agent for the treatment of vascular proliferative diseases, because it effectively and simultaneously influences proliferation, migration, and apoptosis of SMCs. Unfortunately, many substances that were shown to affect SMC cell cycle entry, proteasome function, or retention of lipids and proteoglycans, thereby blocking neointima formation in animal models, failed to show beneficial effects in the clinical setting [7, 26, 33, 37]. However, as WP1066 is a very potent inhibitor with a remarkable long-term inhibitory effect on the phosphorylation of STAT3 in vivo, this compound holds promise to be effective in the clinical setting as well to prevent restenosis when locally applied using drug-eluting stents or balloons.

## Electronic supplementary material

Below is the link to the electronic supplementary material.

Supplementary material 1 (PPTX 12981 kb)
