# Supplementary material for: Inhibition of STAT3 signaling prevents vascular smooth muscle cell proliferation and neointima formation
Source: Basic Res Cardiol. 2012 Mar 15;107(3):261. doi: 10.1007/s00395-012-0261-9 (PMC3350628; doi:10.1007/s00395-012-0261-9)
Supplement: Supplementary file 1 — Supplementary material 1 (PPTX 12981 kb) [file 395_2012_261_MOESM1_ESM.pptx]

## Slide 1
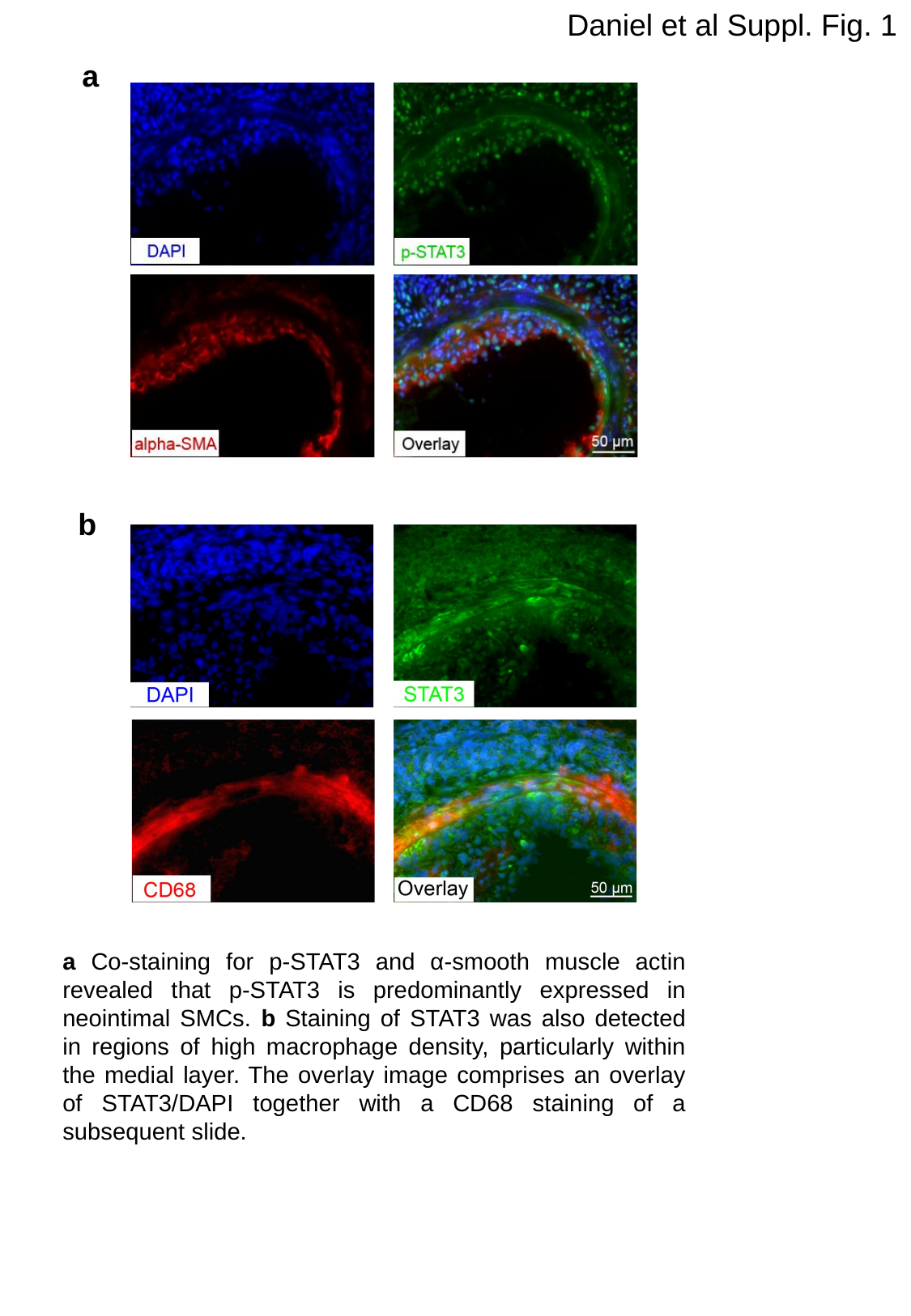

Daniel et al Suppl. Fig. 1
a
b
a Co-staining for p-STAT3 and α-smooth muscle actin revealed that p-STAT3 is predominantly expressed in neointimal SMCs. b Staining of STAT3 was also detected in regions of high macrophage density, particularly within the medial layer. The overlay image comprises an overlay of STAT3/DAPI together with a CD68 staining of a subsequent slide.

## Slide 2
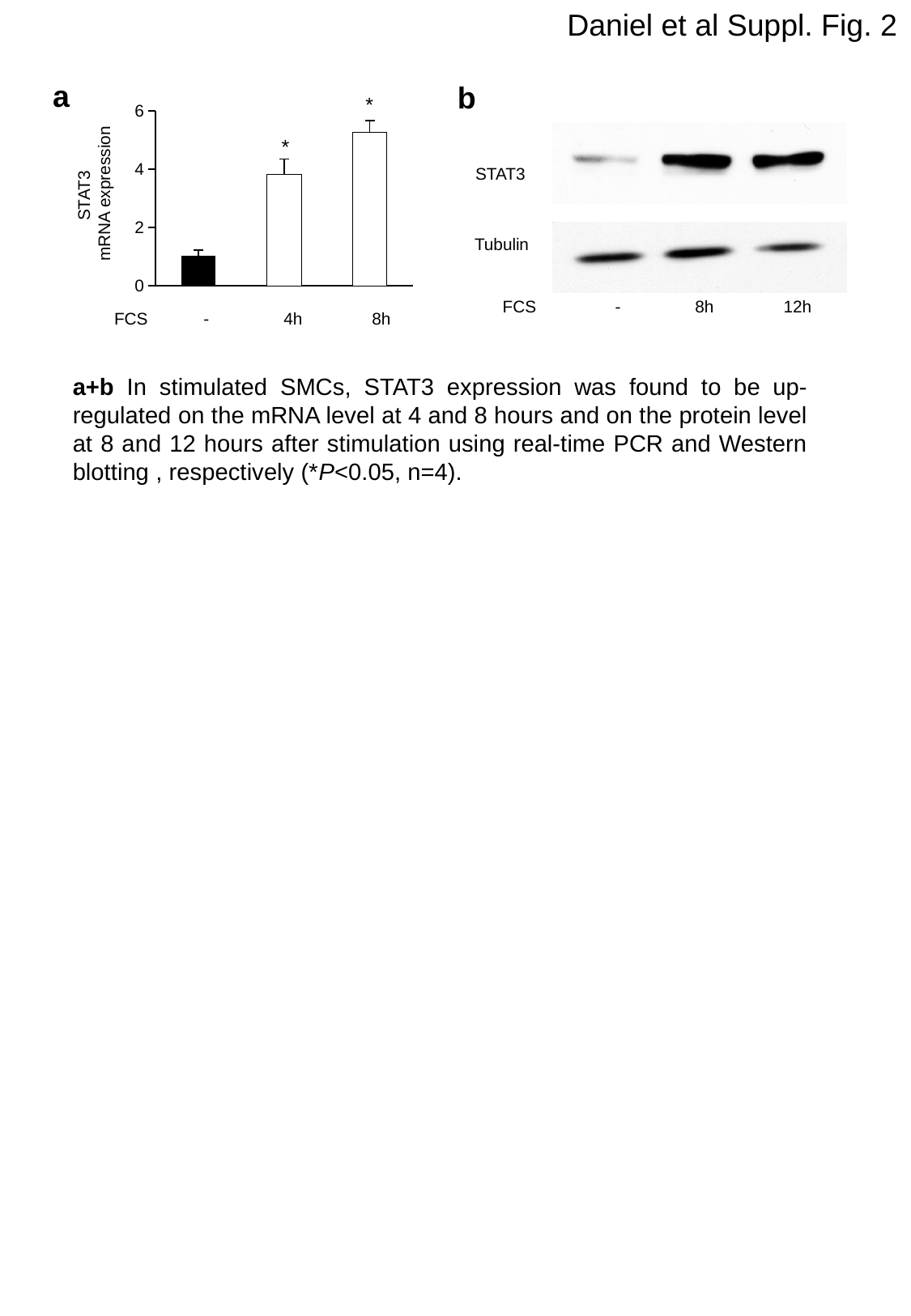

Daniel et al Suppl. Fig. 2
a
b
*
### Chart
| Category | |
|---|---|
*
STAT3
STAT3
mRNA expression
Tubulin
FCS - 8h 12h
FCS - 4h 8h
a+b In stimulated SMCs, STAT3 expression was found to be up-regulated on the mRNA level at 4 and 8 hours and on the protein level at 8 and 12 hours after stimulation using real-time PCR and Western blotting , respectively (*P<0.05, n=4).

## Slide 3
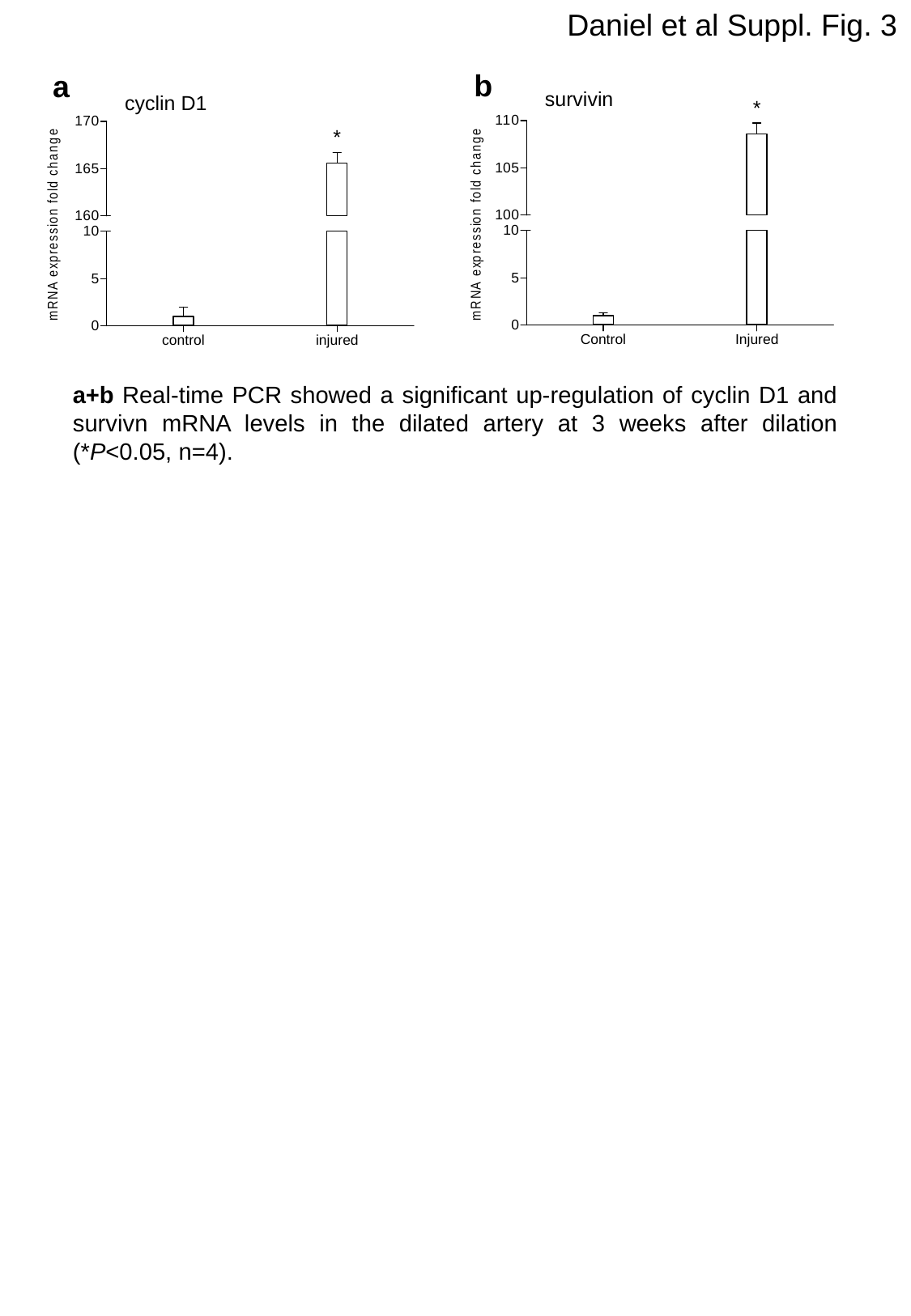

Daniel et al Suppl. Fig. 3
b
a
survivin
cyclin D1
*
*
a+b Real-time PCR showed a significant up-regulation of cyclin D1 and survivn mRNA levels in the dilated artery at 3 weeks after dilation (*P<0.05, n=4).

## Slide 4
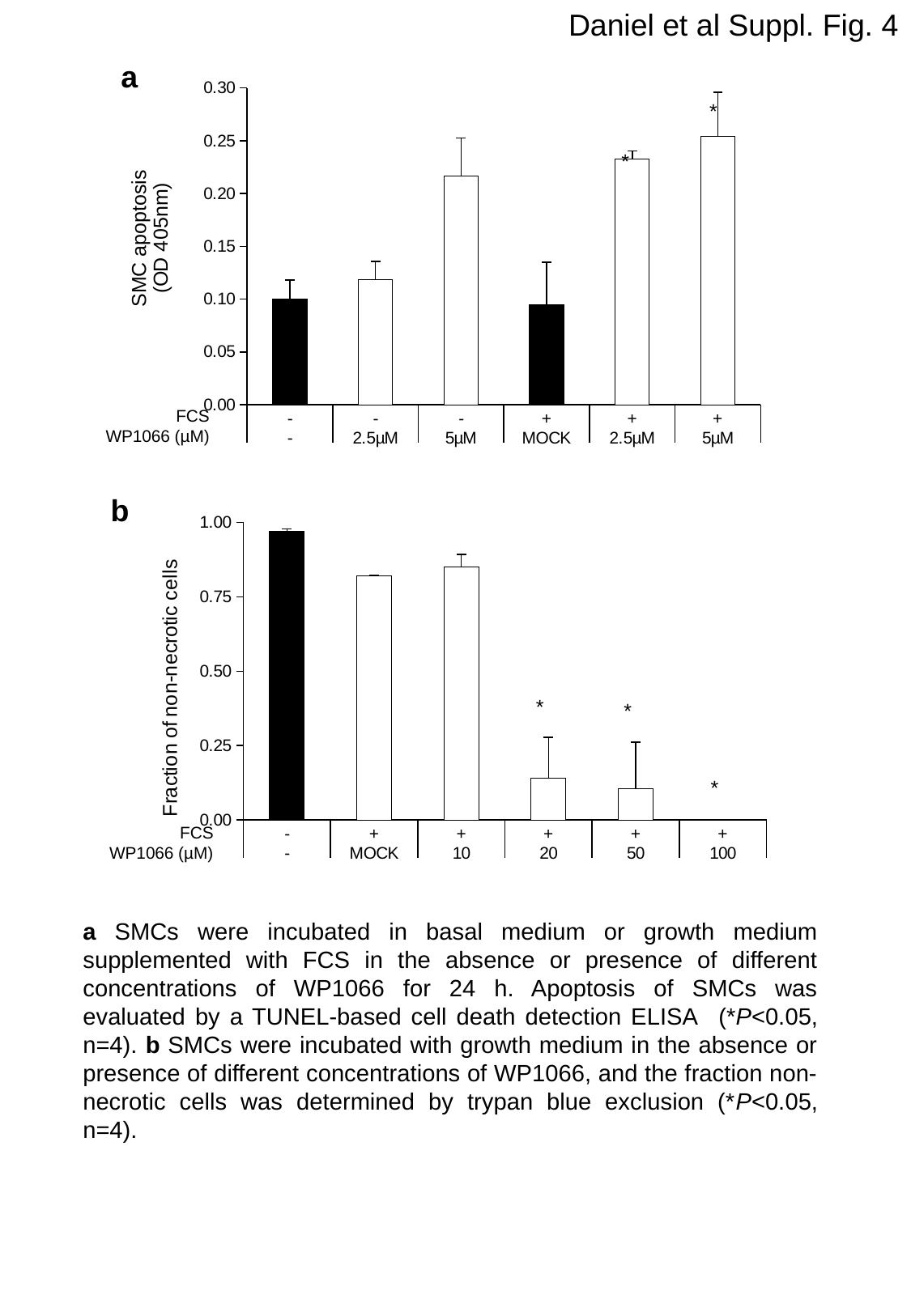

Daniel et al Suppl. Fig. 4
a
### Chart
| Category | |
|---|---|
| - | 0.10016666666666708 |
| - | 0.11816666666666709 |
| - | 0.21650000000000016 |
| + | 0.09483333333333349 |
| + | 0.23283333333333417 |
| + | 0.2541666666666673 |FCS
WP1066 (µM)
*
*
b
### Chart
| Category | |
|---|---|
| - | 0.9691558441558457 |
| + | 0.820490620490621 |
| + | 0.8518518518518526 |
| + | 0.138888888888889 |
| + | 0.105833333333333 |
| + | 0.0 |*
*
*
FCS
WP1066 (µM)
a SMCs were incubated in basal medium or growth medium supplemented with FCS in the absence or presence of different concentrations of WP1066 for 24 h. Apoptosis of SMCs was evaluated by a TUNEL-based cell death detection ELISA (*P<0.05, n=4). b SMCs were incubated with growth medium in the absence or presence of different concentrations of WP1066, and the fraction non-necrotic cells was determined by trypan blue exclusion (*P<0.05, n=4).

## Slide 5
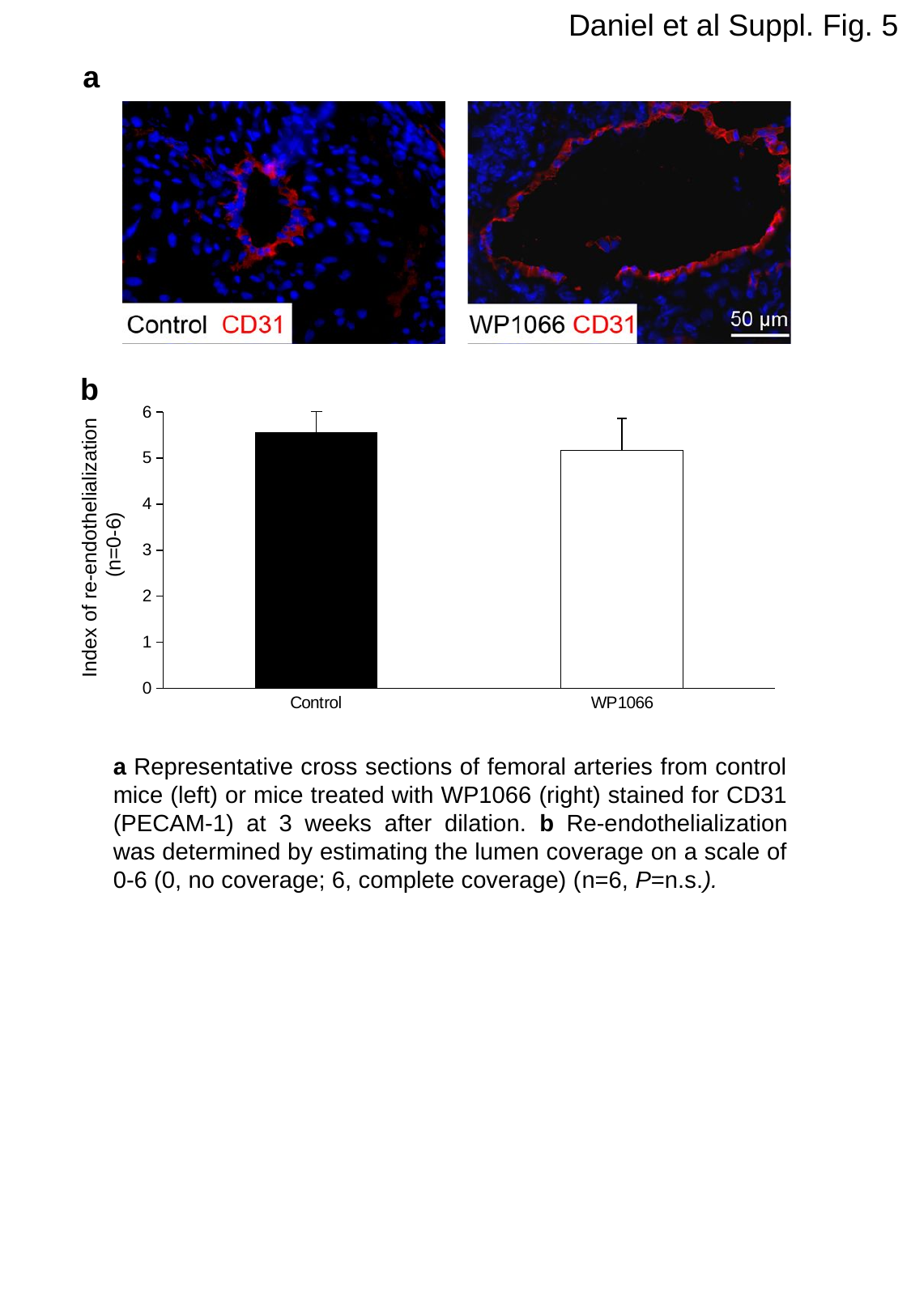

Daniel et al Suppl. Fig. 5
a
b
### Chart
| Category | |
|---|---|
| Control | 5.5555555556111065 |
| WP1066 | 5.166666666666667 |Index of re-endothelialization
(n=0-6)
a Representative cross sections of femoral arteries from control mice (left) or mice treated with WP1066 (right) stained for CD31 (PECAM-1) at 3 weeks after dilation. b Re-endothelialization was determined by estimating the lumen coverage on a scale of 0-6 (0, no coverage; 6, complete coverage) (n=6, P=n.s.).

## Slide 6
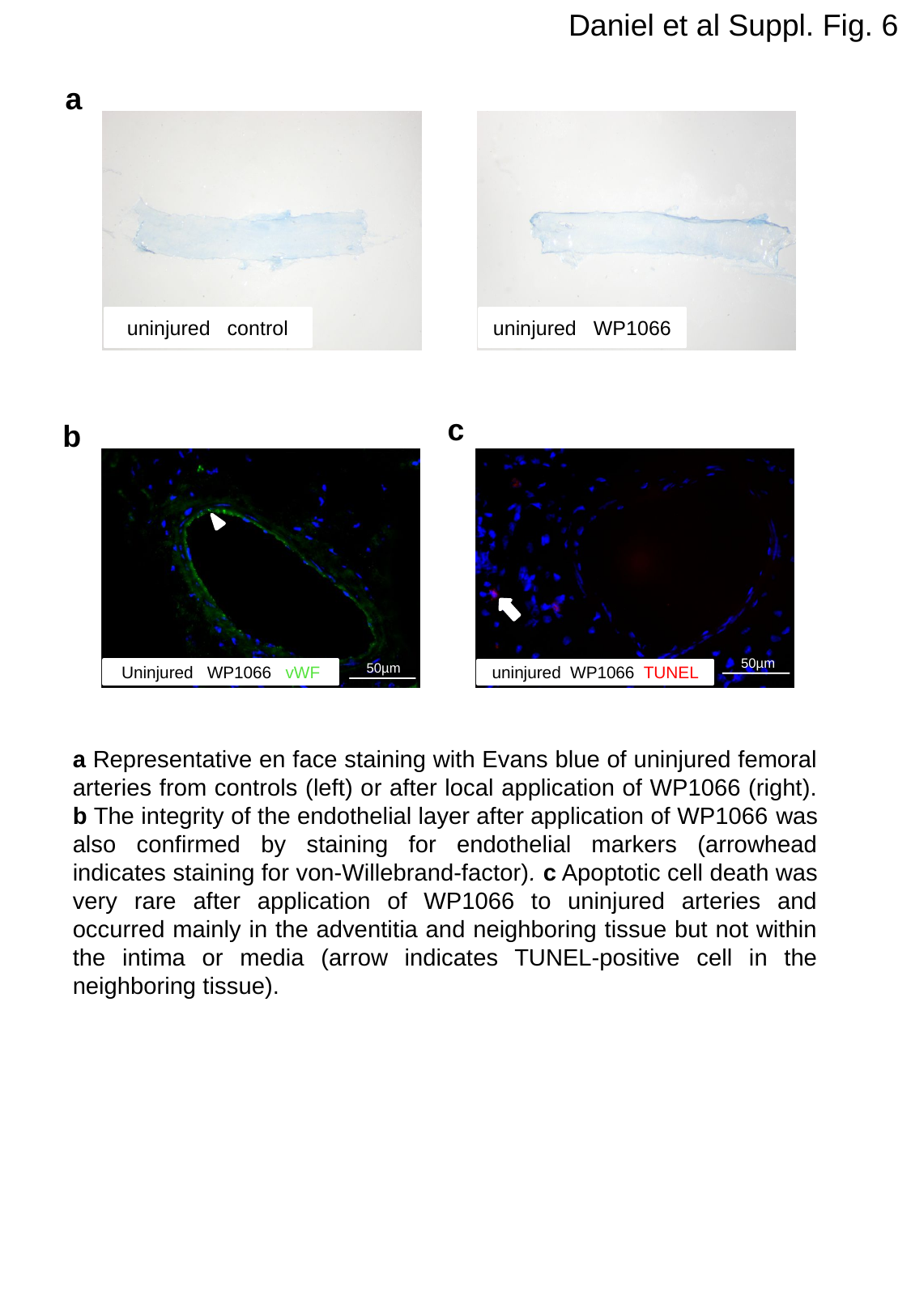

Daniel et al Suppl. Fig. 6
a
uninjured control
uninjured WP1066
c
b
50µm
50µm
Uninjured WP1066 vWF
uninjured WP1066 TUNEL
a Representative en face staining with Evans blue of uninjured femoral arteries from controls (left) or after local application of WP1066 (right). b The integrity of the endothelial layer after application of WP1066 was also confirmed by staining for endothelial markers (arrowhead indicates staining for von-Willebrand-factor). c Apoptotic cell death was very rare after application of WP1066 to uninjured arteries and occurred mainly in the adventitia and neighboring tissue but not within the intima or media (arrow indicates TUNEL-positive cell in the neighboring tissue).

## Slide 7
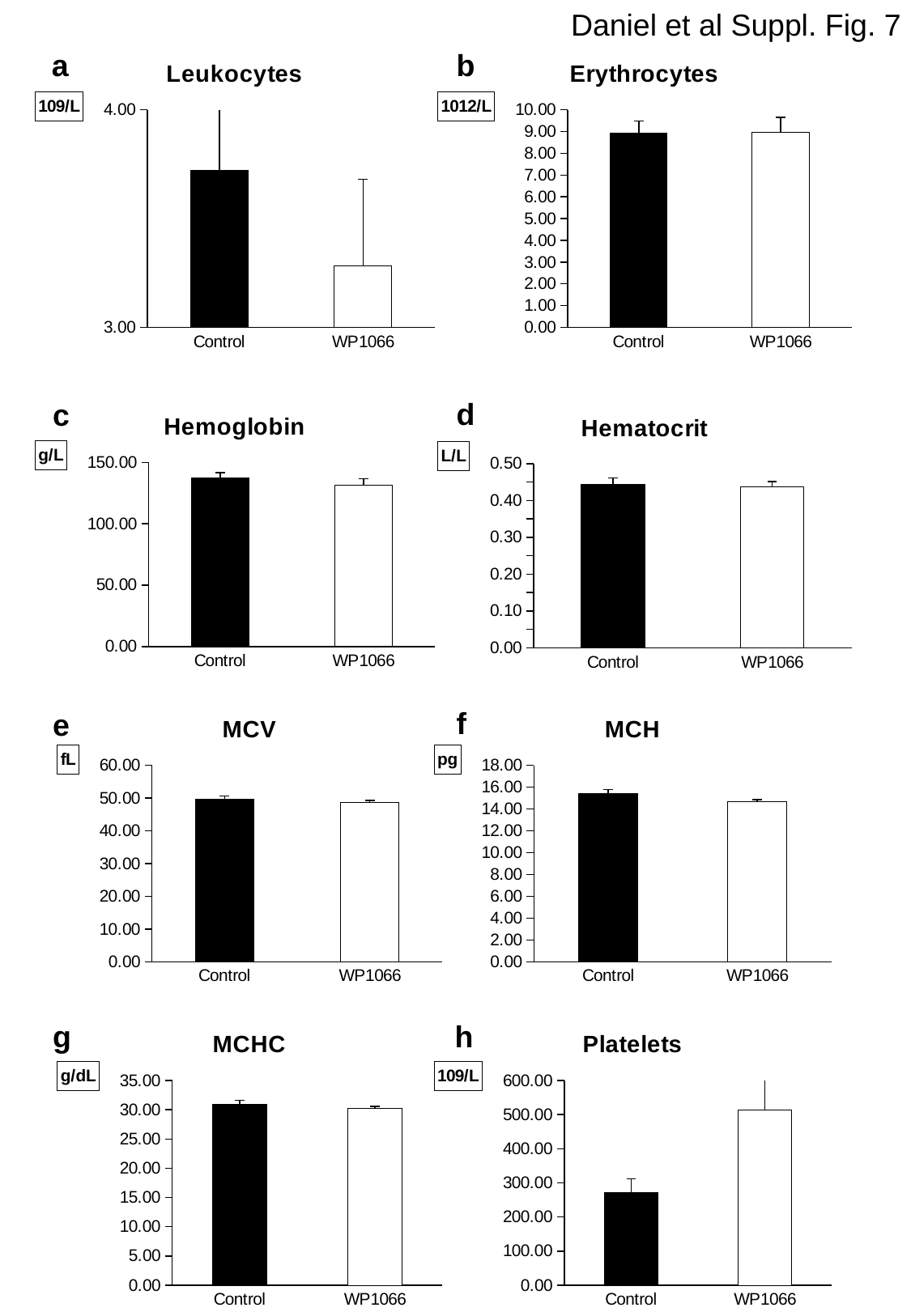

Daniel et al Suppl. Fig. 7
### Chart: Leukocytes
| Category | |
|---|---|
| Control | 3.72 |
| WP1066 | 3.283333333333341 |a
b
### Chart:
| Category | |
|---|---|
| Control | 8.940000000000001 |
| WP1066 | 8.966666666666727 |d
c
### Chart:
| Category | |
|---|---|
| Control | 137.6 |
| WP1066 | 131.66666666666634 |
### Chart:
| Category | |
|---|---|
| Control | 0.444 |
| WP1066 | 0.4366666666666685 |
### Chart:
| Category | |
|---|---|
| Control | 49.6 |
| WP1066 | 48.66666666666636 |
### Chart:
| Category | |
|---|---|
| Control | 15.4 |
| WP1066 | 14.68333333333333 |f
e
g
### Chart:
| Category | |
|---|---|
| Control | 30.959999999999987 |
| WP1066 | 30.16666666666667 |
### Chart:
| Category | |
|---|---|
| Control | 272.2 |
| WP1066 | 513.1666666666666 |h

## Slide 8
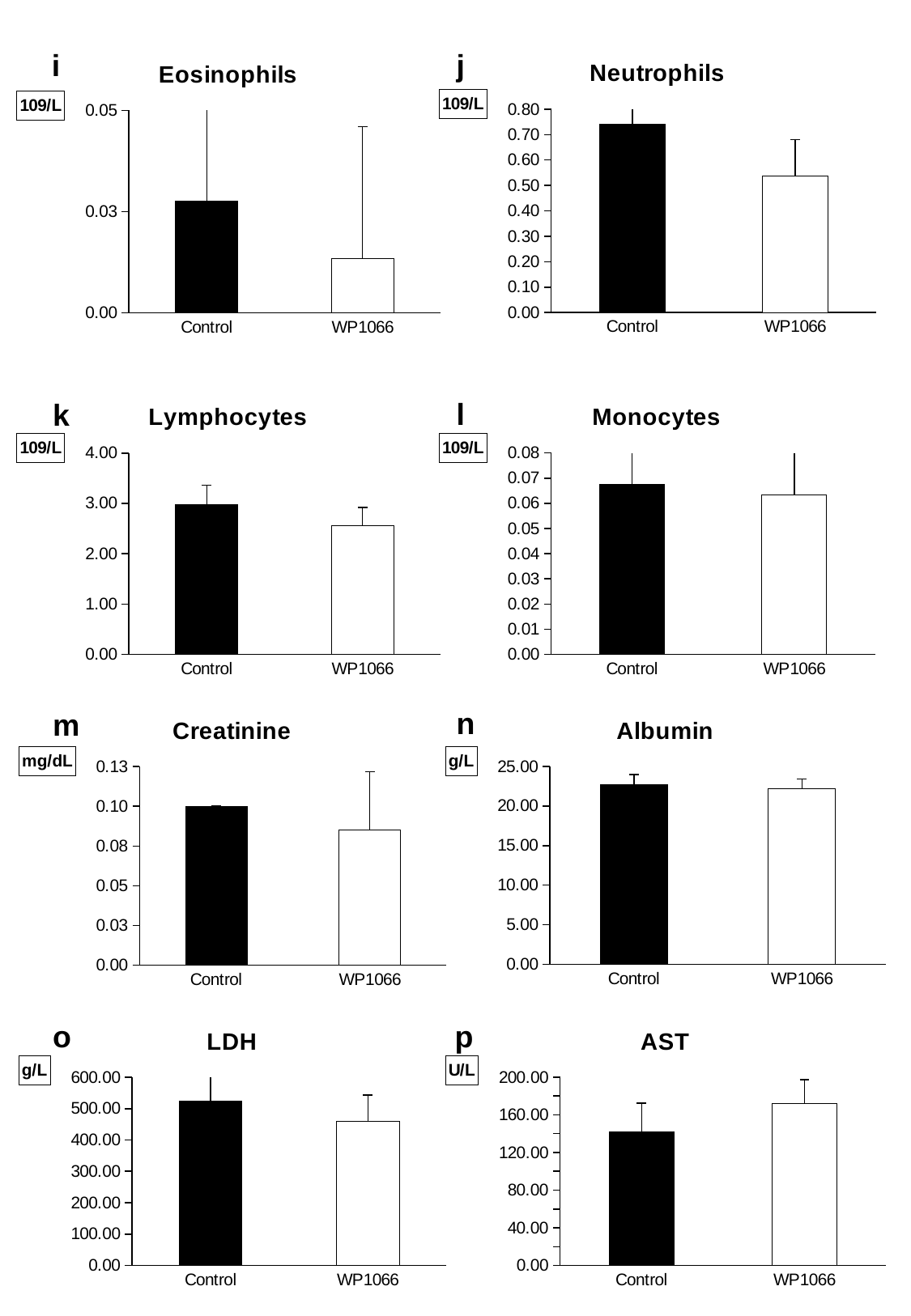

i
### Chart:
| Category | |
|---|---|
| Control | 0.7400000000000027 |
| WP1066 | 0.536666666666667 |j
### Chart:
| Category | |
|---|---|
| Control | 0.02750000000000001 |
| WP1066 | 0.013333333333333303 |
### Chart:
| Category | |
|---|---|
| Control | 2.9775 |
| WP1066 | 2.5566666666666578 |
### Chart:
| Category | |
|---|---|
| Control | 0.0675 |
| WP1066 | 0.0633333333333337 |l
k
### Chart:
| Category | |
|---|---|
| Control | 0.1 |
| WP1066 | 0.08500000000000005 |n
### Chart:
| Category | |
|---|---|
| Control | 22.75 |
| WP1066 | 22.16666666666667 |m
### Chart:
| Category | |
|---|---|
| Control | 523.0 |
| WP1066 | 458.4 |
### Chart:
| Category | |
|---|---|
| Control | 142.0 |
| WP1066 | 172.16666666666634 |o
p

## Slide 9
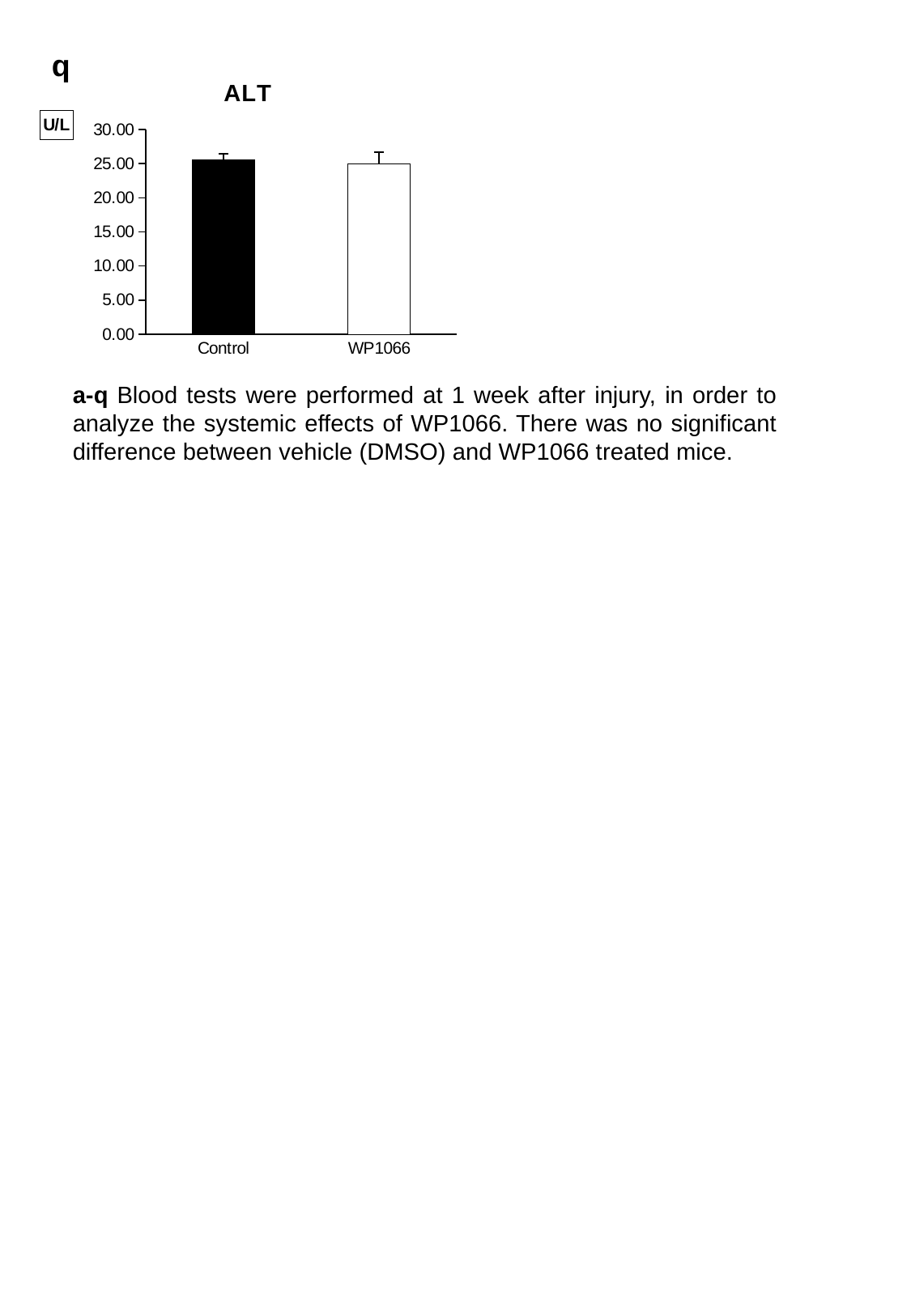

q
### Chart:
| Category | |
|---|---|
| Control | 25.5 |
| WP1066 | 25.0 |a-q Blood tests were performed at 1 week after injury, in order to analyze the systemic effects of WP1066. There was no significant difference between vehicle (DMSO) and WP1066 treated mice.
